# Deciphering the growth stage specific bioactive diversity patterns in *Murraya koenigii* (L.) Spreng. using multivariate data analysis

**DOI:** 10.3389/fpls.2022.963150

**Published:** 2022-08-25

**Authors:** Reetu Verma, Nageswer Singh, Maharishi Tomar, Rakesh Bhardwaj, Dibyendu Deb, Anita Rana

**Affiliations:** ^1^Division of Crop Improvement, ICAR-Indian Grassland and Fodder Research Institute, Jhansi, India; ^2^Department of Chemistry and Biochemistry, Chaudhary Sarwan Kumar Himachal Pradesh Agriculture University, Palampur, HP, India; ^3^Division of Seed Technology, Indian Council of Agricultural Research-Indian Grassland and Fodder Research Institute, Jhansi, India; ^4^Germplasm Evaluation Division, National Bureau of Plant Genetic Resources, New Delhi, India; ^5^Division of Social Science, Indian Council of Agricultural Research-Indian Grassland and Fodder Research Institute, Jhansi, India

**Keywords:** *Murraya koenigii*, developmental stages, multivariate data analysis, HCA, PCA, heat map, phenols, antioxidant

## Abstract

The study was undertaken to characterize the total phenolics, flavonoids, essential oils, quinones, tannins and antioxidant activity of 15 samples of wild *Murraya koenigii* (L.) Spreng. (MK) leaves obtained from different locations of Himachal Pradesh at various growth stages. The results indicated a significant variation in total phenolic content which ranged from [(170.09 ± 4.59 to 303.57 ± 7.94) in pre-flowering, (266.48 ± 7.49 to 450.01 ± 11.78) in the flowering stage, and (212.72 ± 5.37 to 363.85 ± 9.79) in fruiting stage], expressed as mg tannic acid equivalents (TAE)/g. The total flavonoid content ranged from [(15.17 ± 0.36 to 33.40 ± 0.81) in pre-flowering, (25.16 ± 0.67 to 58.17 ± 1.52) in flowering stage, and (17.54 ± 0.42 to 37.34 ± 0.97) in fruiting stage], expressed as mg catechin equivalent (CE)/g. Total tannin content ranged from [(75.75 ± 1.69 to 143 ± 3.74) in pre-flowering, (116 ± 3.26 to 207 ± 5.42) in the flowering stage, and (47 ± 1.18 to 156 ± 4.05) in fruiting stage], expressed as mg TAE/g. The essential oil content ranged from (0.64 ± 0.01 to 0.89 ± 0.02%) in pre-flowering, (0.85 ± 0.02 to 1 ± 0.02%) in flowering stage, and (0.54 ± 0.01 to 0.7 ± 0.01%) in fruiting stage. Quinones ranged from [(2.05 ± 0.05 to 2.97 ± 0.07) in pre-flowering, (3.07 ± 0.07 to 4.95 ± 0.13) in flowering stage, and (1.02 ± 0.02 to 1.96 ± 0.04) in fruiting stage], expressed as mM/min/g tissue. Antioxidant activity ranged from [(4.01 ± 0.09 to 7.42 ± 0.17) in pre-flowering, (8.08 ± 0.19 to 13.60 ± 0.35) in flowering stage, and (3.11 ± 0.06 to 6.37 ± 0.15) in fruiting stage], expressed as μg/ml. Data was subjected to multivariate analysis using principal component analysis (PCA), hierarchical clustering analysis (HCA). This was used for elucidating the intricate relationships between the phytochemical properties. All evaluated phytochemical parameters significantly increased during the growth transition from pre-flowering to the flowering stage, followed by their gradual decrease during the fruiting stage. The present study can serve as rationale for commercializing MK for aromatic and phytopharmaceutical industries.

## Introduction

Earth is a plant-oriented planet and plants are adding value to earth’s diversity and are fundamental to all life (Grygorieva et al., 2021). They have been used for food, medicine or aesthetic purposes for centuries. Using novel plant-based natural products for preventing, treating diseases and enhancing health has recently gained significant scientific attention (Binici et al., 2021). Traditional plant-based medicines have a long history since the ancient civilization where plant-based materials were used as the primary ingredient for drug synthesis (Chandra and Nagani, 2009). An ethnomedicinal plant *Murraya koenigii* (L.) Spreng. or Curry-leaf tree displays distinct bioactivities and medicinal properties. These properties can enable its use in the food, cosmetic, and pharmaceutical industries (Verma et al., 2013; Rajendran et al., 2014; Suthar et al., 2022).

The *Murraya koenigii* (L.) Spreng. (MK) leaves are moderately bitter, slightly pungent and mildly acidic in nature. These leaves are nutritionally rich owing to the numerous phytochemicals, bioactives, essential oils and nutritionally relevant biomolecules (Abeysinghe et al., 2021). The MK leaves are used as appetizers, digestives, analgesics, and anthelmintics in Indian cooking (Bhandari, 2012; Desai et al., 2012). Modern research has now highlighted the potential of MK leaves as a potent antidiabetic (Yadav et al., 2002; Adebajo et al., 2005; Kesari et al., 2005), antifungal (Murugan et al., 2013), nephroprotective (Mahipal and Pawar, 2017), nitric oxide scavenging (Gul et al., 2012), antioxidant (Bhatt et al., 2020), anti-inflammatory, antibacterial (Rautela et al., 2018), anti-cancer (Satyavarapu et al., 2020), immuno-modulatory (Gurjar and Pal, 2021), antihypertensive (An et al., 2020) anti-obesity (Kumar et al., 2020) and lipid lowering agent (Sandamali et al., 2020).These unique medicinal properties of MK leaves are accredited to their diverse metabolites. These include carbazole alkaloids, flavonoids, terpenoids, carbohydrates, phenolics, carotenoids, vitamins, essential oils, tannins, quinones, nicotinic acid, etc. (Balakrishnan et al., 2020).

A unique integration between environmental factors (temperature, photoperiod), soil type, and growth stage can alter the plant phenotype and its phytochemical composition, consequently changing the depot of bioactive compounds present in the MK leaves (Verma et al., 2012; Jimoh et al., 2019a). Also, variation in the germination response of MK to soil type suggests that mineral composition, water relation, and soil texture determines the plant growth, primarily the quantity and diversity of phytochemicals produced by the plant (Yang et al., 2018). Generally, for medicinal plants like MK, metabolic changes during the three growth stages (pre-flowering, flowering, and fruiting) and those caused by the environment can redirect the induction, attenuation, synthesis and transport of various bioactive phytochemicals (phenolics, tannins, antioxidant activity, essential oils, quinones, flavonoids etc.), consequently changing the complete phytochemical profile (Verma and Shukla, 2015). These variations can be viewed as a strategy imparting the plant an ability to adapt and thrive in the changing environments and for establishing a stable ecological relationship between the plant and other microorganisms (Metlen et al., 2009).

Multivariate data analysis (MVDA) is an important statistical approach to efficiently manage complex data (Granato et al., 2018). MVDA delineates the simultaneous analysis of one or more outcome variables (dependent variables) against two or more input variables (independent variables; Hidalgo and Goodman, 2013). It helps to determine the variable dimensions, which is the intricate relationships between several variables contemplated together. Pertinent usage of MVDA can appropriately assess the complex relationships between the phytochemical properties of the assessed sample. It also helps to decipher the similitudes and dissimilarities between multiple sample types, their biochemical characteristics, growth stages, region of cultivation or for gauging the samples in a two-/three-dimensional factor-plane, based on their distinct characteristics (Keithley et al., 2009). Techniques for pattern recognition like hierarchical cluster analysis (HCA) and principal component analysis (PCA) are primarily exploratory data analysis tools that are used for enhancing our comprehension of the data structure and can assess the distribution and association between phytochemicals and their place of cultivation (Zielinski et al., 2018).

Even though the phytochemistry of MK leaves have been investigated in the past, the information on this crop is very scanty and dispersed, especially for those popularly grown in the Indian subcontinent. There is however no report on the phytochemical composition of MK leaves at three different growth stages (pre-flowering, flowering, and fruiting) obtained from different elevations and the use of MVDA tools for deciphering the phytochemical diversity. Thus, the current investigation was performed to characterize the total phenolics, flavonoids, essential oils, quinones, tannins and antioxidant activity of 15 wild samples of MK leaves obtained from different locations of Himachal Pradesh at various growth stages using MVDA.

## Materials and methods

### Sample collection and preparation

**Figure 1:**
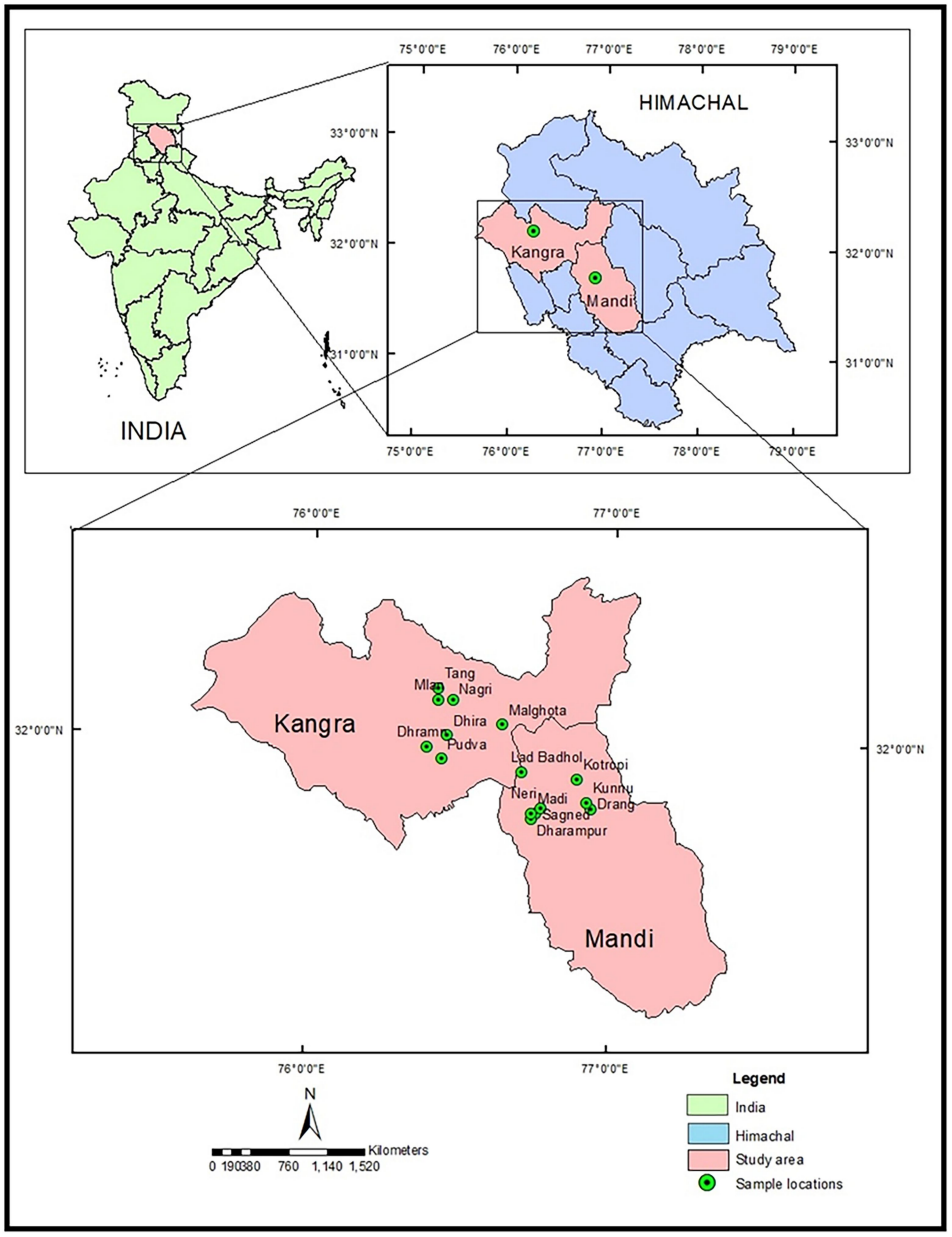
Map of Himachal Pradesh showing study area and sample collection sites.

MK fresh leaves were harvested at three different stages *viz.* pre-flowering, flowering and fruiting stage representing 15 different locations of Kangra and Mandi Districts of Himachal Pradesh. The locations include Pudva, Dhramn, Dhira, Mlan, Malghota, Tang, Nagri, Sagned, Dharampur, Lad Badhol, Neri, Madi, Drang, Kunnu, and Kotropi. The areas explored lies between (31°47′–32°50′ N and 76°25′–76°56′ E and 689–1,339 m altitudinal range). Standard practice and procedures for collecting the samples were followed. Mostly, the local plants were the target of the collection. Random and bulk sampling methods were followed as per the population/quantity of available germplasm material. Passport data, *viz.* location of the site, i.e., village, tehsil, district, latitude, longitude, and altitude were recorded at the different locations (Table 1; Figure 1). After harvesting, the leaf samples were cleaned and divided in two parts. One part was wrapped in aluminum foil, kept in airtight bags and then stored at −20°C. These samples were further used for essential oil yield quantification. The other part was kept for drying in an oven at 60°C–70°C till constant weight. These temperatures do not cause any significant loss of plant phytochemicals. About 10 g of oven dried samples were ground into fine powder followed by manual sieving by using a sieve size of 250 mm and were further utilized for estimation of total phenols, flavonoids, quinones, tannins, and antioxidant activity.

**Table 1:** Details of MK leaf collection sites from different location of Himachal Pradesh.

Sr. no.	Location types	Tehsil, District	Altitude range (m asl)	Latitude/longitude
1	Pudva	Palampur, Kangra	709 m	31°57′N/76°26′E
2	Dhramn	Jaisinghpur, Kangra	807 m	31°59′N/76°32′E
3	Dhira	Dhira, Kangra	946 m	32°01′N/76°27′E
4	Mlan	NB, Kangra	960 m	32°07′N/76°25′E
5	Malghota	Baijnath, Kangra	1,047 m	32°03′N/76°38′E
6	Tang	Dharamshala, Kangra	1,069 m	32°09′N/76°25′E
7	Nagri	Palampur, Kangra	1,278 m	32°07′N/76°28′E
8	Sagned	Sarkaghat, Mandi	689 m	31°48′N/76°45′E
9	Dharampur	Dharampur, Mandi	751 m	31°47′N/76°44′E
10	Lad Badhol	Lad Badhol, Mandi	777 m	31°55′N/76°42′E
11	Neri	Jogindernagar, Mandi	827 m	31°49′N/76°46′E
12	Madi	Sandhol, Mandi	1,016 m	31°48′N/76°44′E
13	Drang	Mandi, Mandi	1,121 m	31°49′N/76°^°^56′E
14	Kunnu	Padar, Mandi	1,201 m	32°50′N/76°55′E
15	Kotropi	Padar, Mandi	1,339 m	31°54′N/76°53′E

MK, *Murraya koenigii* (L.) Spreng.

### Total phenolic content

The phenolic content in MK leaf samples was estimated according to the Folin–Ciocalteu method as described by Julkunen-Tiitto (1985) using a tannic acid standard. Briefly, 200 mg of dried powder in triplicate were taken in test tubes containing 10 ml of 70% acetone and extracted at 37°C for 2 h using a shaking water bath. Subsequently, the tubes were cooled to room temperature and centrifuged at 1,000 g for 20 min. The resulting supernatant obtained was filtered, collected in fresh tubes and used as an extract for estimating total phenols. Hundred μl of sample extract in triplicates was then taken in different test tubes containing 900 μl distilled water. Concurrently blank was prepared in separate tubes by adding 1 ml distilled water. For standard preparation, tannic acid with different concentrations (0.005, 0.01, 0.015, 0.020, 0.025, 0.030, 0.035, 0.040, 0.045, and 0.05 mg) was added to a separate set of test tubes and volume was made to 1 ml with distilled water. Further, 0.5 ml of Folin–Ciocalteu reagent (1N) and 2.5 ml of 20 per cent Na_2_CO_3_ (w/v) was added to each of the three sets of test tubes (sample, blank and standards). The test tubes were wrapped in aluminum foil and kept at incubation for 40 min at room temperature. Folin–Ciocalteu reagent is a mixture of molybdates and tungstates and works by the oxidation–reduction reaction. The mixture of molybdates-heteropolyphosphotungsates is reduced by phenolic compounds forming a blue colored chromogen under basic condition by Na_2_CO_3_. The absorbance of the resulting solution (sample, standard) was measured at 725 nm against a blank by using a UV–visible spectrophotometer. The total phenolic content was computed using a standard curve made from tannic acid and the results are depicted as mg tannic acid equivalents (TAE) per gram dry weight basis (DWB) of MK leaves.

### Total flavonoid content

The total flavonoids content were determined based on the procedure proposed by (Swain and Hillis, 1959) with slight modification. One-gram dried powdered MK leaf samples in triplicate were ground with 10 ml of methanol and the whole content was transferred to the test tubes. The tubes were then agitated constantly for 12 h in a shaking water bath maintained at 37°C. Thereafter, the content was centrifuged for 20 min at 3,500 g. The filtrate was collected in a beaker and dried with the help of a rotary evaporator. The dried extract obtained was re-dissolving in 1 ml of methanol. Briefly, 250 μl of methanol extract was taken in triplicate in test tubes and then 2,250 μl distilled water was added to it. Simultaneously, blank was prepared by adding 2,500 μl distilled water in separate tubes. For the preparation of standard, catechin, 5, 10, 15, 20, 25, 30, and 35 μg was added to a separate set of tubes and the volume was made to 2,500 μl with distilled water. After that 150 μl of 15%, NaNO_3_ solution was added to the reaction mixture (sample, blank and standards) and then incubated for 6 min at ambient temperature. After incubation, 150 μl of 10% AlCl_3_ was added and then 2,000 μl of 4% NaOH and 200 μl of distilled water were added. The solution was mixed well and the absorbance of the reaction mixture (sample, standard) was recorded at 510 nm against blank with the help of a UV–visible spectrophotometer. The total phenolic content was calculated with reference to the catechin standard curve and results are expressed in mg catechin equivalent (CE) per gram DWB of MK leaf powder.

### *In vitro* antioxidant activity assays

This assay determined the potential of MK crude extracts to neutralize the free radical, 2,2-diphenyl-1-picrylhydrazyl (DPPH). DPPH is a stable free radical which has an unpaired valence electron at one atom of nitrogen bridge. The antioxidant activity was determined according to a method by Sharma and Bhat (2009) with slight modifications. Each extract in triplicates and catechin standard (20, 40, 60, 80, 100, 120, and 140 μl) was taken in a separate set of test tubes, subsequently, 3.0 ml of methanol and 1.0 ml of DPPH was added to it. A combination of 3.0 ml of methanol and 1.0 ml of DPPH in a separate test tube was used as a control. The three sets of a tube (sample, standard, and control) were kept in dark at 30°C for 30 min. The absorbance of each solution (sample, standard and control) was measures at 517 nm using methanol as blank. Finally, the percentage of DPPH free radical scavenging activity (per cent inhibition) of MK extracts was calculated according to Eq (1)


(1) $$\begin{array}{c} \%\mathrm{DPPH inhibition}=\frac{\left(\mathrm{Absorbance control}-\mathrm{Absorbance sample}\right)}{\mathrm{Absorbance control}}\\ \;\times 100 \end{array}$$

IC_50_ value (to measure the antioxidant amount needed to reduce the free radical DPPH concentration by 50%) for each solution was computed from the regression line generated from the graph of per cent DPPH inhibition against the concentration of each solution using Eq (2)


(2) $${IC}_{50}\mathrm{value}\mu g/\mathrm{ml}=\frac{\left(50-y\;\mathrm{intercept}\right)}{\mathrm{slope}}$$

### Essential oil yield

Essential oil yield was determined through a method by Yadegarinia et al. (2006) using the Clevenger apparatus. Eighty-gram fresh leaf of each sample in triplicate was placed in a 1,000 ml round bottom flask and subjected to hydro-distillation for 6 h. The oil collected was dried over a hot water bath and preserved at 4°C for further analysis. Essential oil yield (%) was determined using Eq (3):


(3) $$\begin{array}{c} \mathrm{Essential oil yield}\%=\frac{\mathrm{Mass of essential oil obtained}\;\left(g\right)}{\mathrm{Mass of fresh leaf samples}\;\left(g\right)}\\ \;\times 100 \end{array}$$

### Quinones

Quinones were estimated through a method by Mahadevan and Sridhar (1986). Briefly, 1.0 g sample in triplicate were ground with chilled 10 ml 0.1 M sodium phosphate buffer (pH 6.6) using pestle and mortar. The mixture was then centrifuged at 1,000 g for 30 min at 4°C. The supernatant was collected in fresh tubes and was used as an extract. Accurately 1.5 ml extract in triplicate was taken in a test tube. Then 3.0 ml phosphate buffer and 3.0 ml standard catechol were added to it. The mixture was vortexed gently and incubated in a water bath for 1 h at 60°C. Nearly 2.0 ml sample was withdrawn in a test tube in duplicates. Four ml of Trichloroacetic acid (without ascorbic acid) was added to one test tube and 4.0 ml of trichloroacetic acid (containing ascorbic acid) to another. The precipitate was filtered and the absorbance of each solution was measured at 400 nm against a blank. The results were expressed as mM/min/g tissue DWB.

### Total tannin content

The total tannin content of MK samples was estimated by the Folin–Denis method (Pansera et al., 2003) with slight modifications. Briefly, 200 mg of dried powder in triplicate were taken in test tubes containing 10 ml of 70% acetone and extracted at 37°C for 2 h using a shaking water bath. Subsequently, the tubes were cooled to room temperature and centrifuged at 1,000 g for 20 min. The resulting supernatant obtained was filtered, collected in fresh tubes and used as an extract for estimating total tannin content. Briefly, 400 μl of extract in triplicate was taken in test tubes and 400 μl of Folin–Denis reagent was added to it. After 3 min, 400 μl of 8% Na_2_CO_3_ solution was added to these test tubes. The solution was mixed properly and then incubated for 1 h. The solution was centrifuged for 5 min at 1,000 g and the absorbance of each solution was taken at 725 nm against blank. For the preparation of standard, tannic acid (12.5, 25, 50, 100, 500, and 1,000 μg) was used and the results are expressed as mg tannic acid equivalents (TAE) per gram DWB of MK leaves.

### Statistics

ArcGIS 10.1 platform (ESRI Inc., United States) was used for preparing the map for the study area and sample location sites. Unless stated otherwise all the analysis was performed as triplicates. Statistical significance was set at a 95% confidence level. Hierarchical cluster analysis (HCA) was done by Ward clustering algorithm using squared Euclidean distance by a trial version of IBM SPSS. The correlation between total phenolics, flavonoids, essential oils, quinones, tannins and antioxidant activities were assessed using Pearson’s correlation coefficient. The correlation analysis was performed using Jamovi version 1.2.27 at a 5% level of significance. The differences between various phytochemical attributes were statistically assessed by one-way analysis of variance (ANOVA). The differences among means were evaluated by *post-hoc* test (Duncan’s multiple range test) at (*p* < 0.05) level using a trial version of IBM SPSS. The data were subjected to principal component analysis (PCA) for assessing the phytochemical constituents in MK leaves that determine the observed variation and visualize the tentative relationships among these components using Jamovi version 1.2.27. To maximize the loading of a parameter in the component axis and for interpreting the pattern of a specific parameter, the orthogonal varimax rotation was applied to PCA. The heatmap for relative composition and Pearson’s correlation coefficient was constructed using a web interface (MetaboAnalyst; Chong et al., 2019).

## Results and discussion

### Multivariate data analysis

To enhance our understanding of the data organization and structure, its classification and distribution, PCA and HCA were used for visualizing the phytochemical composition of wild MK leaves collected from 15 different locations of Himachal Pradesh, India, at different growth stages, *viz.* pre-flowering, flowering, and fruiting stages.

#### Hierarchical clustering analysis

Hierarchical clustering analysis (HCA) is a statistical exploratory analysis technique that is used for differentiating sample groups or individual variables using similarity or density measurements. HCA was used to explore the sample organization into individual groups, and among groups, delineating their hierarchy. The results of the HCA are represented through a dendrogram, which shows the association between samples in form of a tree. In a typical dendrogram, the samples are joined through branches with corresponding similarity values. Similarities present in the traits of these samples are determined by an inverse function of their inter-distance. Various matrices are used for assessing the distance between two samples in a specific multivariate space, most common being Mahalanobis distance and Euclidean distance. In the present study, Ward’s linkage was used as the linkage criterion, which is based on the optimal values of a target function(Granato et al., 2015) and Euclidean distance was used as the adequate linkage benchmark and sample distance metric. Also, the samples were agglomeratively clustered, where each sample was initially regarded as a cluster and later, the cluster pairs were merged.

**Figure 2:**
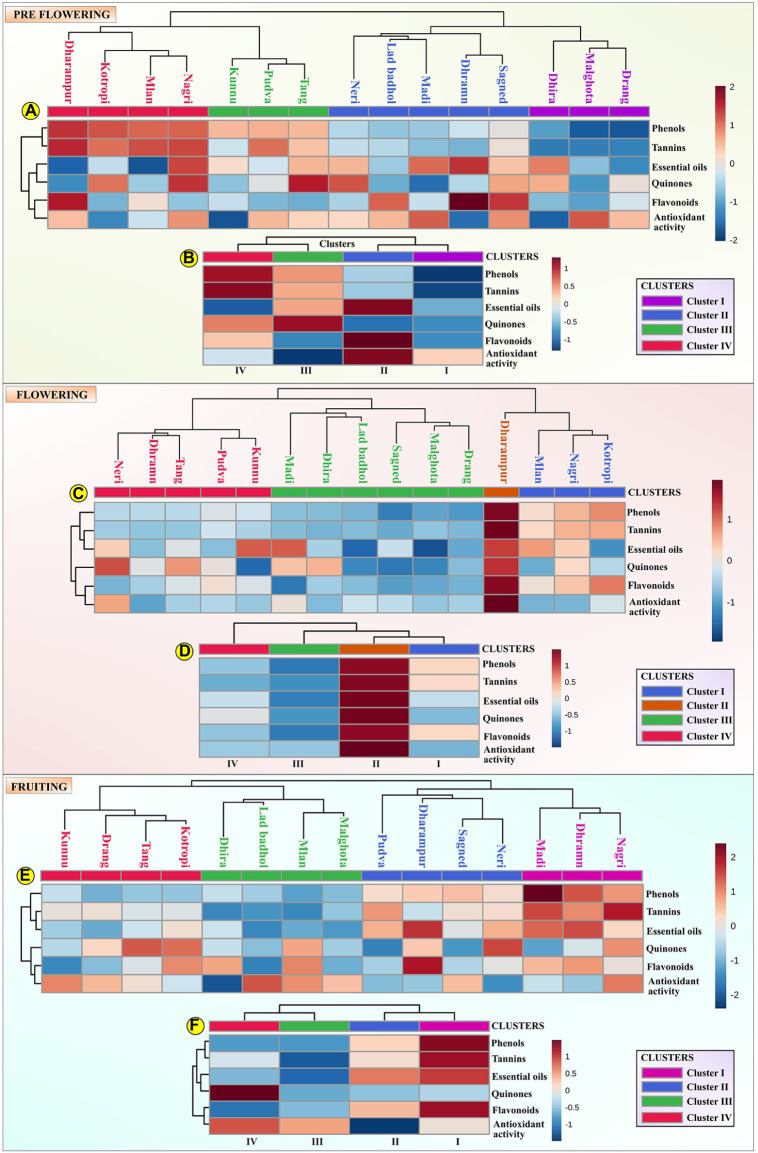
The assessed samples were organized into four different clusters, depicting their hierarchy and presented as a dendrogram and scaled phytochemical attributes are represented through a heat map. **(A)** represents the hierarchical cluster integrated with a heat map representing the scaled phytochemical attributes for each sample at the pre-flowering stage. The data is scaled between +2 and −2; **(B)** represents the hierarchical cluster integrated with a heat map representing the mean scaled value of each cluster at the pre-flowering stage. The data is scaled between +1 and –1; **(C)** represents the hierarchical cluster integrated with a heat map representing the scaled phytochemical attributes for each sample at the flowering stage. The data is scaled between +1 and −1; **(D)** represents the hierarchical cluster integrated with a heat map representing the mean scaled value of each cluster at the flowering stage. The data is scaled between +1 and −1; **(E)** represents the hierarchical cluster integrated with a heat map representing the scaled phytochemical attributes for each sample at the fruiting stage. The data is scaled between +2 and −2; **(F)** represents the hierarchical cluster integrated with a heat map representing the mean scaled value of each cluster at the fruiting stage. The data is scaled between +1 and −1.

The HCA was performed at each stage of MK leaf development (*viz.* pre-flowering, flowering and fruiting). The leaf samples were grouped into four clusters for each of the development stages. The mean value of the phytochemical composition for each cluster for every growth stage is indicated in Table 2 and is depicted by Box-and-whisker plots in Supplementary Figures S1–S3. The hierarchical clusters along with the scaled relative phytochemical composition is depicted through heat maps in Figures 2A–F.

**Table 2:** Mean values of phytochemicals in MK leaves organized into different clusters at each growth stage.

Pre-flowering stage				
Biochemical trait	Cluster I (*N* = 3)	Cluster II (*N* = 5)	Cluster III (*N* = 3)	Cluster IV (*N* = 4)
Phenols	180.32 ± 16.566^a^	225.55 ± 11.244^b^	265.75 ± 1.395^c^	294.5 ± 6.451^d^
Flavonoids	17.34 ± 2.531^a^	25.62 ± 6.586^a^	17.22 ± 1.845^a^	21.42 ± 6.552^a^
Antioxidant activity	6.04 ± 1.722^a^	6.3 ± 1.204^a^	5.67 ± 1.444^a^	5.95 ± 1.042^a^
Essential oil yield	0.75 ± 0.089^a^	0.82 ± 0.061^a^	0.79 ± 0.03^a^	0.73 ± 0.112^a^
Tannins	76.25 ± 0.661^a^	97.2 ± 8.228^b^	117.33 ± 15.011^c^	137.5 ± 4.509^d^
Quinones	2.45 ± 0.26^a^	2.44 ± 0.334^a^	2.58 ± 0.351^a^	2.55 ± 0.374^a^
**Flowering stage**				
Biochemical trait	Cluster I (*N* = 3)	Cluster II (*N* = 1)	Cluster III (*N* = 6)	Cluster IV (*N* = 5)
Phenols	371.25 ± 19.968^c^	450.01 ± 9.046^d^	287.86 ± 12.938^a^	324.14 ± 7.617^b^
Flavonoids	44.35 ± 4.498^c^	58.17 ± 1.04^d^	30.21 ± 2.948^a^	36.38 ± 3.839^b^
Antioxidant activity	8.75 ± 0.69^a^	13.6 ± 0.75^b^	9.09 ± 0.541^a^	9.16 ± 1.119^a^
Essential oil yield	0.93 ± 0.047^a,b^	1 ± 0^b^	0.9 ± 0.05^a^	0.93 ± 0.038^a,b^
Tannins	159 ± 7^c^	207 ± 2.14^d^	119.9 ± 2.431^a^	128 ± 5.568^b^
Quinones	3.8 ± 0.346^a^	4.95 ± 0.89^b^	3.65 ± 0.561^a^	4.09 ± 0.666^a^
**Fruiting stage**				
Biochemical trait	Cluster I (*N* = 3)	Cluster II (*N* = 4)	Cluster III (*N* = 4)	Cluster IV (*N* = 4)
Phenols	328.51 ± 32.465^c^	275.68 ± 7.234^b^	229.75 ± 14.332^a^	231.48 ± 11.091^a^
Flavonoids	29.32 ± 2.433^a^	27.34 ± 6.816^a^	25.29 ± 7.423^a^	24.23 ± 6.067^a^
Antioxidant activity	5.05 ± 0.926^a^	4.46 ± 0.775^a^	5.24 ± 1.459^a^	5.39 ± 0.532^a^
Essential oil yield	0.67 ± 0.032^b^	0.65 ± 0.037^b^	0.56 ± 0.026^a^	0.59 ± 0.026^a^
Tannins	142.33 ± 14.572^c^	101.5 ± 16.603^b^	54.19 ± 10.767^a^	93 ± 3.651^b^
Quinones	1.45 ± 0.325^a^	1.43 ± 0.449^a^	1.41 ± 0.225^a^	1.68 ± 0.27^a^

MK, *Murraya koenigii* (L.) Spreng.; Data are expressed as Mean ± Standard Deviation; Means with different superscript within the same row for each cluster show significant difference at *p* ≤ 0.05; *N*, Number of samples lying in each cluster; phenols were expressed as mg tannic acid equivalents (TAE)/g; flavonoids as mg catechin equivalent (CE)/g; antioxidant activity as μg/ml; essential oils as %; tannins as mg TAE/g; quinones as mM/min/g tissue.

For the pre-flowering stage, cluster I was characterized by samples having lower phenol content (180.32 ± 16.56 mg TAE/g) and tannin (76.25 ± 0.66 mg TAE/g) content. Cluster II was characterized by samples with higher content of essential oils (0.82% ± 0.061%), antioxidant activity (6.3 ± 1.20 μg/ml) and flavonoids (25.62 ± 6.58 mg CE/g). Cluster III is indicated by samples with higher content of quinones (2.58 ± 0.35 mM/min/g tissue). Cluster IV is characterized by germplasm with higher content of phenol content (294.5 ± 6.45 mg TAE/g), tannins (137.5 ± 4.50 mg TAE/g) and lower content of essential oils (0.73 ± 0.11%). For the flowering stage, cluster I was represented by samples having lower antioxidant activity. Cluster II was characterized by samples having higher content of phenol content (450.01 ± 9.04 mg TAE/g), flavonoids (58.17 ± 1.04 mg CE/g), antioxidant activity (13.6 ± 0.75 μg/ml), essential oil (1% ± 0%), tannins (207 ± 2.14 mg TAE/g), and quinones (4.95 ± 0.89 mM/min/g tissue). Cluster III was characterized by samples having lower content of phenols (287.86 ± 12.93 mg TAE/g), flavonoids (30.21 ± 2.94 mg CE/g), antioxidant activity (9.09 ± 0.54 μg/ml), essential oil (0.9% ± 0.05%), tannins (119.9 ± 2.43 mg TAE/g), and quinones (3.65 ± 0.56 mM/min/g tissue). For fruiting stage, cluster I was characterized by samples having a higher content of flavonoids (29.32 ± 2.43 mg CE/g), essential oils (0.67 ± 0.03%), and tannins (142.33 ± 14.57 mg TAE/g). The cluster II was displayed by samples having a lower antioxidant activity (4.46 ± 0.77 μg/ml). Cluster III was depicted by germplasms having lower content of phenols (229.75 ± 14.33 mg TAE/g), tannins (54.19 ± 10.76 mg TAE/g), essential oil (0.56% ± 0.02%), and quinones (1.41 ± 0.22 mM/min/g tissue). Cluster IV was indicated by germplasms with higher content of quinones (1.68 ± 0.27 mM/min/g tissue), antioxidant activity (5.39 ± 0.53 μg/ml) and lower content of flavonoids (24.23 ± 6.0 mg CE/g).

#### Principal component analysis

The phytochemical composition of MK leaves for pre-flowering, flowering and fruiting stages were scaled and subjected to factor analysis using principal component analysis (PCA). This was done to distinguish the traits that determine the observed variability in the composition and visualize the potential associations among these traits. PCA algorithms investigate the direction having the maximum variation within a multidimensional data space. This is based on the postulation that the maximum variability (depicted by a high value of variance) equates to more information. To discern the accuracy of this direction, the data matrix was mean-centered column-wise, ascertaining that the axes rotate about the data centroid. The rotating axis explaining the highest variance delineates the first (lowest-order) principal component (PC1). The second axis resides in the direction with the maximum variance noncorrelated (orthogonal) among all directions with respect to PC1. Subsequently, the second PC (PC2) explains all possible information that is not explained by PC1 and so on for other PCs. The PCs substantiate the same information obtained from the assessed parameters but exhibits the additional advantage of being uncorrelated reciprocally, circumventing any copious information between the parameters (Granato et al., 2018). PCs can be regarded as a linear amalgamation of original variables, with each multiplied with a weight coefficient, known as loading. Through a geometrical perspective, loadings portray the cosine values of angle between PCs and the original variables. These loading values range between −1 and + 1, insinuating their role in defining a specific PC. Greater is the cosine value, lesser is the rotational angle between PC and the variable, nearer are the two directions, higher is the contribution of that variable to the given PC. PCA has a distinct ability to display the lengthy and complex information present in the data through bi and tridimensional plots (biplots, score plots, and loading plots). Each PC accounts for a part of the variation in the data is determined through Eigenvalue (Dobriban, 2017). Every Eigenvalue accords to an Eigenvector, determining the variance among the associated PCs. Factors with Eigenvalues > 1.0 were seen in agreement with Kaiser’s criteria (Kaiser, 1960).

Before subjecting the data to component analysis, Kaiser-Meyer-Olkin (KMO) Measure of Sampling Adequacy (MSA) and Bartlett’s test for sphericity was used as the main assumption checks. Bartlett’s test juxtaposes the identity matrix to the observed correlation matrix (Tobias and Carlson, 1969). It helps to identify the possible redundancy among variables that can be reiterated by a small number of factors. This test validates whether or not the variables in the data are correlated in such a way that they can be potentially outlined by a smaller set of factors. The null hypothesis for this test is that the variables are uncorrelated or orthogonal. The alternate hypothesis states that the variables are sufficiently correlated to where the correlation matrix significantly diverges from the identity matrix. Thus if Bartlett’s test is significant at (*p* < 0.5), the correlation matrix is significantly divergent from the null hypothesis, indicating that the data reduction through PCA can meaningfully compress the data. The values of Bartlett’s test for our data were *p* < 0.001 for phenols, flavonoids, antioxidant activity, essential oils, tannins, and quinones (Supplementary Table S1).

The KMO is another statistical test that determines the suitability of data for factor analysis through PCA. It assesses the variance proportion of the variables that could result from the underlying factors. Lesser is the proportion of variation, more is the suitability of the data for PCA (Lestari, 2021). Values ranging between 0.9 and 1.0 are contemplated as excellent for subjecting the data to PCA. The values between 0.7 and 0.9 are regarded as good and those between 0.5 and 0.7 are adequate. If the values are lower than 0.5, the PCA results will be unable to provide any meaningful interpretation. The KMO values in the present study ranged from 0.665 to 0.850 (Supplementary Table S2), showing the high appropriateness of the data for PCA.

Following the determination of the representative PCs on the basis of explained variance and sample grouping/differentiation, the loadings were assessed to elucidate the fundamental association between the data structure. The loading can be contemplated as linear combination coefficients of the initial variables from which the PCs are synthesized. These factor loadings (FLs) are the variable coordinates divided by the square root of the eigenvalue associated with the specific component. FLs having positive values indicate that the factor will lie higher on the positive axis of that PC. In the present study, quinones (FL1: 0.911), essential oils (FL: 0.882), antioxidant activity (FL1: 0.816), tannins (FL2: 0.910), phenols (FL2: 0.809), flavonoids (FL3: 0.902; Supplementary Table S3).

**Figure 3:**
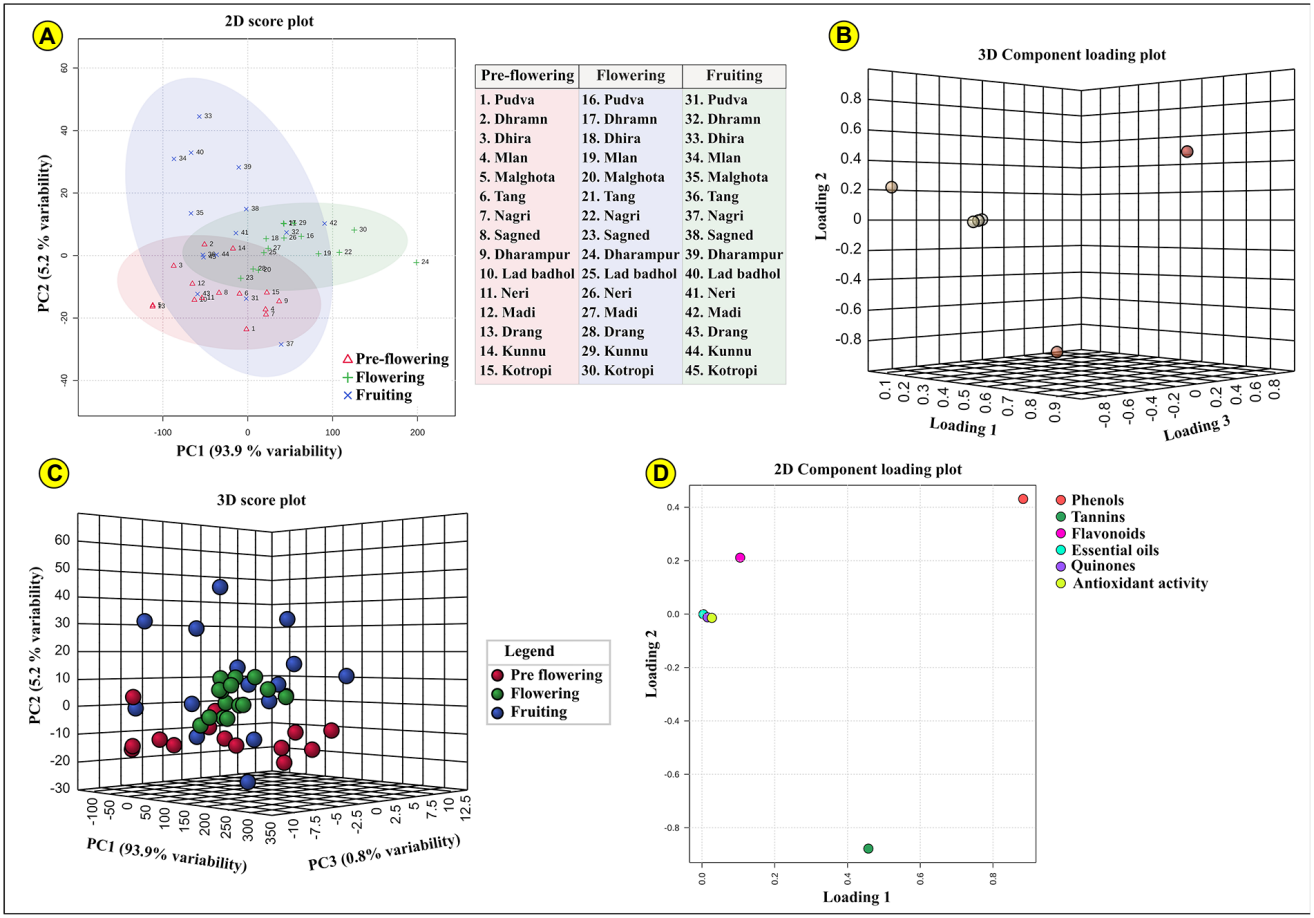
Represents the PCA (Principal component analysis); **(A)** two-dimensional and **(B)** three-dimensional PCA component loading plot for all three developmental stages (pre-flowering, flowering, and fruiting). The samples lying in each coordinate are clearly indicated; **(C)** indicated the three-dimensional PCA score plot; **(D)** indicates two-dimensional component loading plot for each of the assessed phytochemical parameters.

PCA revealed that the first two components explained 99.1% variability in the data, in terms of phenols, flavonoids, antioxidant activity, essential oils, tannins, and quinones. PC1 described 93.9% of the total variation, in which the dominant parameters include quinones, essential oils and antioxidant activity. PC2 explained 5.2% of the total variation in which the chief parameters include phenols and tannins. These determining factors showed high values of FLs and lower uniqueness. Based on the calculated FL values, the 2D and 3D PCA score plots showing the distribution of phytochemicals in ML leaves at three different growth stages is indicated in Figure 3.

### Phytochemical composition of MK leaves and its relation with growth stage

**Figure 4:**
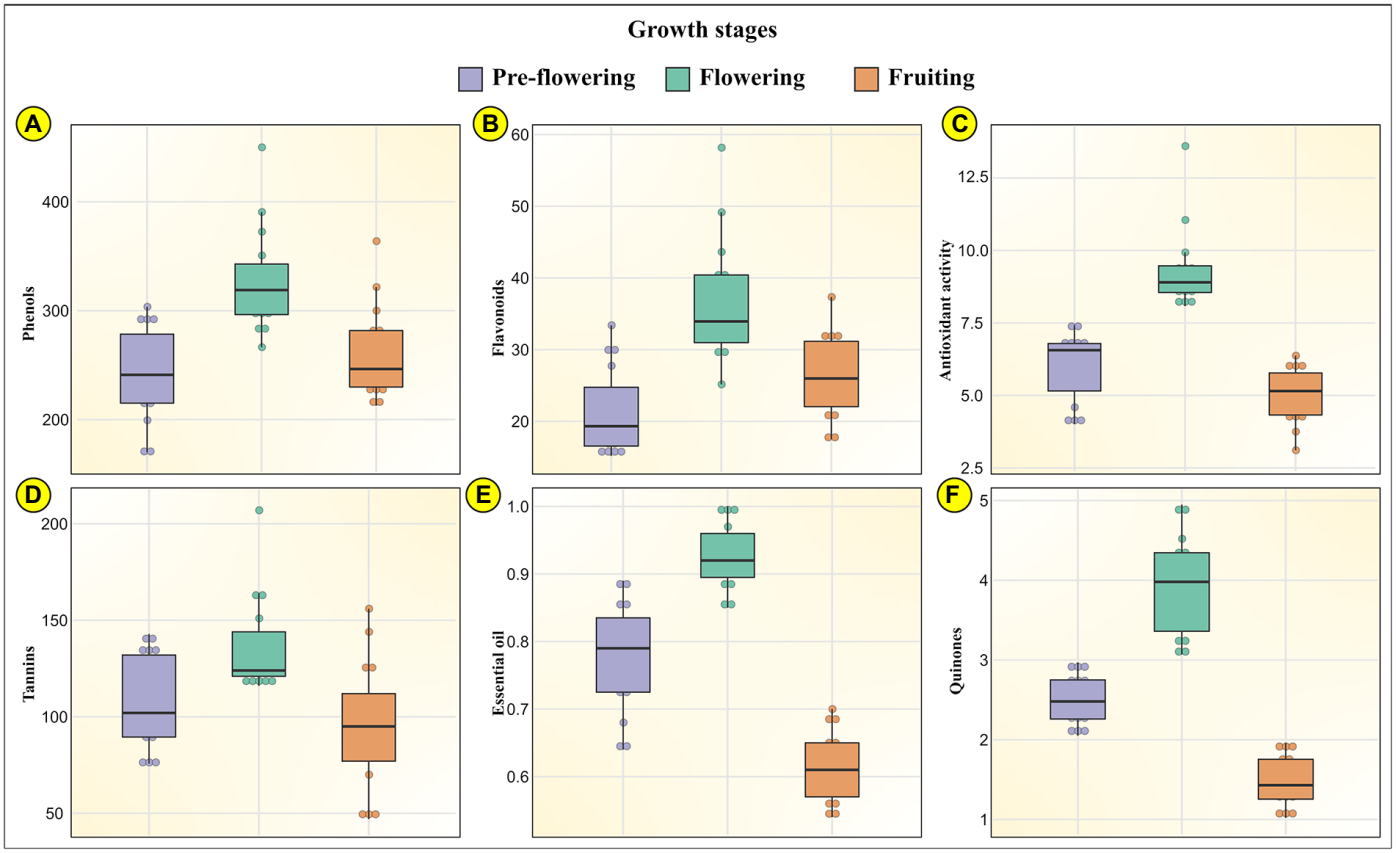
Represents the phytochemical profile of MK (*Murraya koenigii* (L.) Spreng.) leaves through Box and Whisker Plots at the three developmental stages (pre-flowering, flowering, and fruiting) for **(A)** phenols; **(B)** flavonoids; **(C)** antioxidant activity; **(D)** tannins; **(E)** essential oils; and **(F)** quinones.

**Figure 5:**
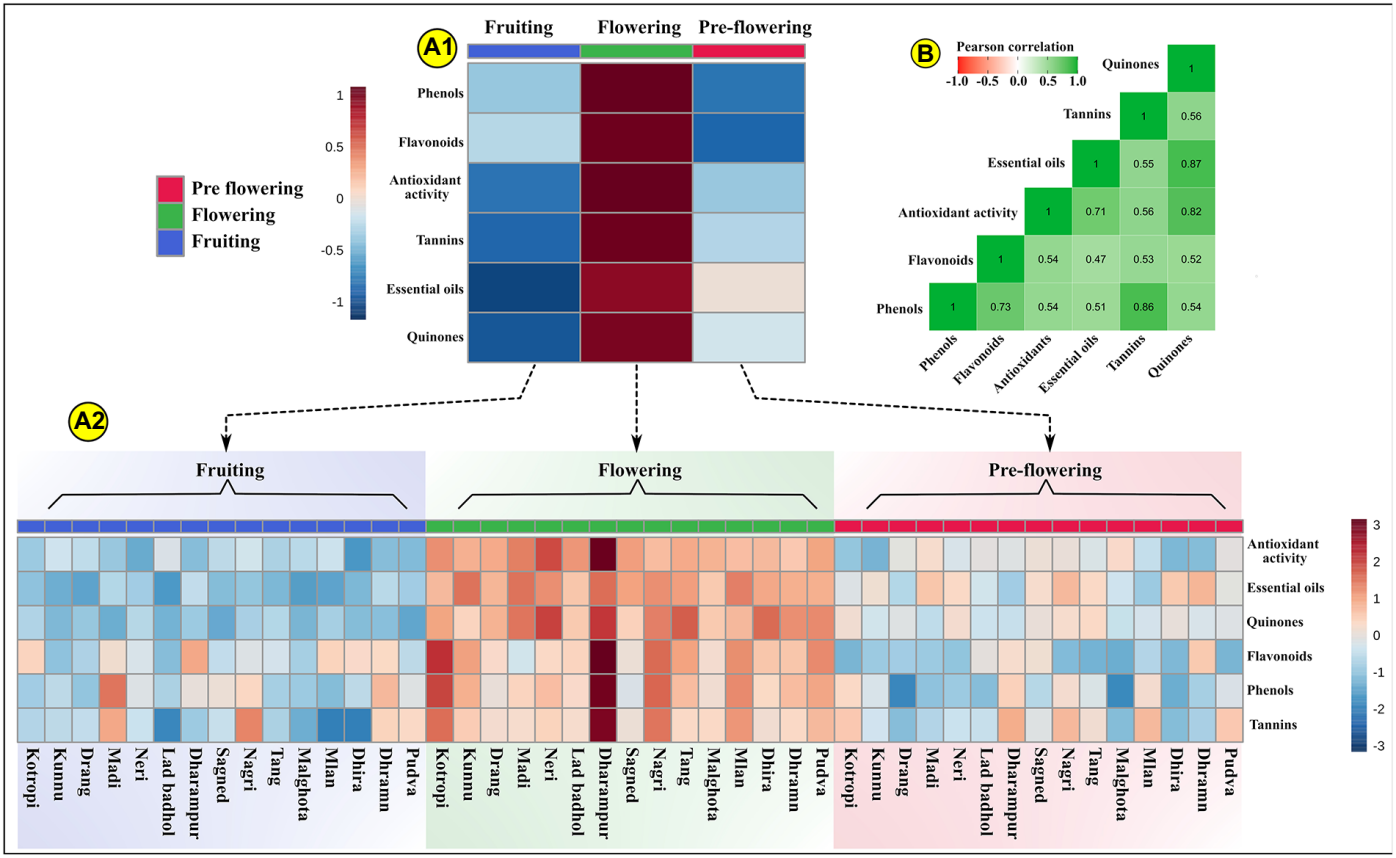
Represents the phytochemical composition of MK (*Murraya koenigii* (L.) Spreng.) leaves through a heat map. **(A1)** Indicates the scaled mean phytochemical profile of MK leaves at three development stages. The data is scaled between +1 and –1. **(A2)** Represents the scaled photochemical profile of MK leaves for each sample at each of the three development stages. The data is scaled between +3 and −3; **(B)** Indicates the Pearson’s r correlation values through a heat map. The Pearson’s r (+1 to −1) is indicated through different color intensities.

Plants synthesize and accumulate a diversity of high and low molecular weight secondary metabolites which exhibit potent bioactivities (Quideau et al., 2011; Kasote et al., 2015). These natural bioactive metabolites belong to distinct chemical groups like flavonoids, phenols, quinones, tannins, diterpenes, isoflavones, anthocyanins (Jimoh et al., 2019b). These phytochemicals display unique overlapping roles as hydrogen donors, singlet oxygen quenchers, reducing agents, plant defense agents against biotic and abiotic stresses, radiation absorbers, allelopathy, pollinator attractors, transition metal chelators (Harborne and Williams, 2000; Demidchik, 2015; do Nascimento et al., 2018). The concentration of these show variation in different plant parts, growth phases, planting seasons (Lagnika et al., 2016). A unique combination of externally provided mineral resources from the soil (place of growth) and developmental stage delineate the carbon-nutrient balance of the plant. This determines the biosynthesis and translocation of these metabolites in the plants (Mahmood et al., 2012). It is therefore critical to assess the phytochemical composition of MK leaves at different growth stages to accurately ascertain the pharmacological potential of the plant. Our results indicated that the growth stages significantly influence the phytochemistry of the plant. Our data indicated that all evaluated phytochemical parameters in MK leaves significantly increased during the growth transition from pre-flowering to flowering stage, followed by a gradual decrease during the fruiting stage (Tanko et al., 2005). This could possibly result from the variation in expression of phenylpropanoid pathway mediating enzymes during different growth stages (Kariñho-Betancourt et al., 2019) and differences in root absorption capacity, soil nutrient availability, climatic conditions, photosynthesis rate, etc. (Briat et al., 2020; Tomar et al., 2021). The mean phytochemical composition of MK leaves is represented through Box and Whisker Plots at all the three developmental stages in Figure 4, by bar graphs in Supplementary Figure S4 and through comprehensive heat maps in Figure 5. Also the phytochemical composition is indicated in Table 3.

**Table 3:** Values of phytochemicals in MK leaves organized into different clusters at each growth stage.

Pre-flowering stage						
Place of collection	Phenols	Flavonoids	Antioxidant activity	Essential oil yield	Tannins	Quinones
Pudva	267.29 ± 6.254^f^	16.39 ± 0.383^a,b^	6.64 ± 0.155^e,f,g^	0.76 ± 0.018^c,d^	132 ± 3.088^f^	2.48 ± 0.058^e^
Dhramn	232.15 ± 5.658^d,e^	33.4 ± 0.814^i^	4.27 ± 0.104^a,b^	0.89 ± 0.022^h^	88 ± 2.145^b^	2.37 ± 0.058^d,e^
Dhira	199.43 ± 4.449^b^	16.73 ± 0.373^c^	4.11 ± 0.092^a^	0.85 ± 0.019^f,g,h^	75.75 ± 1.69^a^	2.68 ± 0.06^f^
Mlan	289.61 ± 7.321^g^	21.76 ± 0.55^f^	5.72 ± 0.145^c^	0.64 ± 0.016^a^	137 ± 3.463^f,g^	2.32 ± 0.059^d^
Malghota	171.43 ± 4.069^a^	15.17 ± 0.36^a^	7.42 ± 0.176^h^	0.72 ± 0.017^b,c^	76 ± 1.804^a^	2.17 ± 0.052^a,b,c^
Tang	265.39 ± 6.273^f^	15.93 ± 0.377^a,b^	6.36 ± 0.15^d,e^	0.82 ± 0.019^e,f,g^	118 ± 2.789^e^	2.97 ± 0.07^h^
Nagri	290.18 ± 7.548^g^	17.11 ± 0.445^b,c^	6.94 ± 0.181^f,g^	0.88 ± 0.023^h^	138 ± 3.59^f,g^	2.92 ± 0.076^g,h^
Sagned	241.07 ± 6.779^e^	29.39 ± 0.826^h^	6.98 ± 0.196^g^	0.81 ± 0.023^e,g^	109 ± 3.065^d^	2.7 ± 0.076^f^
Dharampur	303.57 ± 7.948^g^	30.55 ± 0.8^h^	6.56 ± 0.172^d,e^	0.65 ± 0.017^a^	143 ± 3.744^g^	2.14 ± 0.056^a,b^
Lad badhol	214.15 ± 5.122^c^	27.75 ± 0.664^g^	6.64 ± 0.159^e,f,g^	0.73 ± 0.017^c^	98 ± 2.344^c^	2.23 ± 0.053^b,c,d^
Neri	224.33 ± 5.353^c,d^	18.21 ± 0.435^c,d^	6.26 ± 0.149^d^	0.82 ± 0.02^e,f,g^	100 ± 2.386^c^	2.86 ± 0.068^g,h^
Madi	216.07 ± 5.819^c^	19.35 ± 0.521^d,e^	7.35 ± 0.198^h^	0.86 ± 0.023^g,h^	91 ± 2.451^b^	2.05 ± 0.055^a^
Drang	170.09 ± 4.598^a^	20.12 ± 0.544^e^	6.59 ± 0.178^d,e,f^	0.68 ± 0.018^a,b^	77 ± 2.081^a^	2.51 ± 0.068^e^
Kunnu	264.57 ± 6.557^f^	19.33 ± 0.479^d,e^	4.01 ± 0.099^a^	0.79 ± 0.02^d,e^	102 ± 2.528^c^	2.29 ± 0.057^c,d^
Kotropi	294.64 ± 8.16^g^	16.25 ± 0.45^a,b^	4.59 ± 0.127^b^	0.75 ± 0.021^c,d^	132 ± 3.656^f^	2.8 ± 0.078^f,g^
**Flowering stage**						
	Phenol	Flavonoid	Antioxidant	Essential oil	Tannins	Quinones
Pudva	334.93 ± 7.836^e,f^	40.57 ± 0.949^f^	9.04 ± 0.212^c,d,e,f^	0.9 ± 0.021^a,b,c,d^	137 ± 3.205^d^	4.05 ± 0.095^d^
Dhramn	319.27 ± 7.781^d,e^	33.94 ± 0.827^d^	8.08 ± 0.197^a^	0.9 ± 0.022^a,b,c,d^	123 ± 2.998^a,b,c^	3.98 ± 0.097^d^
Dhira	298.48 ± 6.658^b,c^	33.86 ± 0.755^d^	8.45 ± 0.188^a,b^	0.91 ± 0.02^b,c,d^	118.38 ± 2.641^a^	4.39 ± 0.098^f^
Mlan	350.7 ± 8.865^f^	40.25 ± 1.017^e,f^	8.33 ± 0.211^a,b^	0.97 ± 0.025^e,f^	151 ± 3.817^e^	3.47 ± 0.088^b^
Malghota	286.3 ± 6.796^b,c^	30.15 ± 0.716^b,c^	8.65 ± 0.205^b,c^	0.85 ± 0.02^a^	123 ± 2.919^a,b,c^	3.23 ± 0.077^a^
Tang	318.93 ± 7.538^d,e^	38.14 ± 0.902^e^	8.9 ± 0.21^b,c,d,e^	0.93 ± 0.022^c,d,e^	124 ± 2.931^a,b,c^	4.52 ± 0.107^f^
Nagri	372.46 ± 9.689^g^	43.63 ± 1.135^g^	8.38 ± 0.218^a,b^	0.95 ± 0.025^d,e,f^	162 ± 4.214^f^	4.16 ± 0.108^d,e^
Sagned	266.48 ± 7.493^a^	29.18 ± 0.821^b^	9.23 ± 0.26^d,e,f^	0.92 ± 0.026^c,d,e^	116 ± 3.262^a^	3.14 ± 0.088^a^
Dharampur	450.01 ± 11.782^i^	58.17 ± 1.523^i^	13.6 ± 0.356^i^	1 ± 0.026^f^	207 ± 5.42^g^	4.95 ± 0.13^g^
Lad badhol	294.35 ± 7.041^b,c^	31.95 ± 0.764^c,d^	9.39 ± 0.225^e,f^	0.86 ± 0.021^a,b^	121 ± 2.894^a,b,c^	3.25 ± 0.078^a^
Neri	318.1 ± 7.591^d,e^	31.01 ± 0.74^b,c^	11.05 ± 0.264^h^	0.95 ± 0.023^d,e,f^	127 ± 3.031^b,c^	4.82 ± 0.115^g^
Madi	300.92 ± 8.104^c,d^	25.16 ± 0.678^a^	9.93 ± 0.267^g^	0.99 ± 0.027^f^	121 ± 3.259^a,b,c^	4.3 ± 0.116^e,f^
Drang	280.65 ± 7.586^a,b^	30.95 ± 0.837^b,c^	8.88 ± 0.24^b,c,d,e^	0.89 ± 0.024^a,b,c^	120 ± 3.244^a,b^	3.57 ± 0.097^b,c^
Kunnu	329.46 ± 8.165^e^	38.25 ± 0.948^e^	8.73 ± 0.216^b,c,d^	0.99 ± 0.025^f^	129 ± 3.197^c^	3.07 ± 0.076^a^
Kotropi	390.58 ± 10.817^h^	49.16 ± 1.362^h^	9.55 ± 0.264^f,g^	0.88 ± 0.024^a,b,c^	164 ± 4.542^f^	3.76 ± 0.104^c^
**Fruiting stage**						
	Phenol	Flavonoid	Antioxidant	Essential oil	Tannins	Quinones
Pudva	270.54 ± 6.33^d^	22.79 ± 0.533^c^	4.2 ± 0.098^c^	0.65 ± 0.015^e,f^	124 ± 2.901^g^	1.02 ± 0.024^a^
Dhramn	321.67 ± 7.84^f^	31.45 ± 0.767^f^	4.34 ± 0.106^c^	0.69 ± 0.017^g^	127 ± 3.095^g^	1.43 ± 0.035^f^
Dhira	246.42 ± 5.497^c^	30.88 ± 0.689^e,f^	3.11 ± 0.069^a^	0.6 ± 0.013^c,d^	47.76 ± 1.065^a^	1.38 ± 0.031^e,f^
Mlan	212.72 ± 5.377^a^	32.36 ± 0.818^f^	5.94 ± 0.15^g^	0.56 ± 0.014^a,b^	47 ± 1.188^a^	1.73 ± 0.044^h^
Malghota	225.01 ± 5.341^a,b^	20.39 ± 0.484^b^	5.52 ± 0.131^f^	0.55 ± 0.013^a,b^	70 ± 1.661^b^	1.28 ± 0.03^c,d^
Tang	230.36 ± 5.445^b^	25.52 ± 0.603^d^	5.15 ± 0.122^e^	0.6 ± 0.014^c,d^	89 ± 2.104^c,d^	1.92 ± 0.045^i,j^
Nagri	300.01 ± 7.804^e^	26.67 ± 0.694^d^	6.1 ± 0.159^g,h^	0.63 ± 0.016^d,e^	156 ± 4.058^i^	1.78 ± 0.046^h^
Sagned	283.94 ± 7.984^d^	23.24 ± 0.654^c^	5.56 ± 0.156^f^	0.61 ± 0.017^c,d^	98 ± 2.756^f^	1.09 ± 0.031^a,b^
Dharampur	279.48 ± 7.317^d^	37.34 ± 0.978^g^	4.31 ± 0.113^c^	0.7 ± 0.018^g^	84 ± 2.199^c^	1.63 ± 0.043^g^
Lad badhol	234.83 ± 5.617^d,c^	17.54 ± 0.42^a^	6.37 ± 0.152^h^	0.54 ± 0.013^a^	52 ± 1.244^a^	1.23 ± 0.029^c^
Neri	268.76 ± 6.413^d^	25.98 ± 0.62^d^	3.75 ± 0.089^b^	0.65 ± 0.016^e,f^	100 ± 2.386^f^	1.96 ± 0.047^j^
Madi	363.85 ± 9.799^g^	29.85 ± 0.804^e^	4.72 ± 0.127^d^	0.68 ± 0.018^f,g^	144 ± 3.878^h^	1.13 ± 0.03^b^
Drang	219.65 ± 5.937^a,b^	21.32 ± 0.576^b^	5.61 ± 0.152^f^	0.56 ± 0.015^a,b^	97 ± 2.622^f^	1.58 ± 0.043^g^
Kunnu	246.44 ± 6.107^c^	18.01 ± 0.446^a^	6 ± 0.149^g^	0.58 ± 0.014^b,c^	95 ± 2.354^e,f^	1.34 ± 0.033^d,e^
Kotropi	229.47 ± 6.355^b^	32.08 ± 0.888^f^	4.78 ± 0.132^d^	0.62 ± 0.017^d,e^	91 ± 2.52^d,e^	1.87 ± 0.052^i^

MK, *Murraya koenigii* (L.) Spreng.; Data are expressed as Mean ± Standard Deviation; Means with different superscripts within the same row for each cluster show significant difference at *p* ≤ 0.05. Phenols were expressed as expressed as mg tannic acid equivalents (TAE)/g; flavonoids as mg catechin equivalent (CE)/g; antioxidant activity as μg/ml; essential oils as %; tannins as mg TAE/g; quinones as mM/min/g tissue.

For the pre-flowering stage, the total phenols in MK leaves ranged from 170.09 ± 4.59 mg TAE/g in samples collected from Drang to 303.57 ± 7.94 mg TAE/g in samples obtained from Dharampur. For the flowering stage, the total phenols ranged from 266.48 ± 7.49 mg TAE/g in Sagned samples to 450.01 ± 11.78 mg TAE/g in Dharampur samples. In the fruiting stage, the content ranged from 212.72 ± 5.37 mg TAE/g in Mlan samples to 363.85 ± 9.79 mg TAE/gm in Madi samples. The results corroborated with those of (Sivakumar and Meera, 2013) who indicated the total phenol content of 111.6 ± 3.85 to 532.8 ± 2.81 mg GAE/g, similarly (Kaur et al., 2016) indicated a content of 119.6 mg GAE/g in MK leaves. Also for the pre-flowering stage, the total flavonoid content MK leaves ranged from 15.17 ± 0.36 mg CE/g in Malghota samples to 33.40 ± 0.81 mg CE/g in Dhramn samples. For the flowering stage, the content ranged from 25.16 ± 0.67 mg CE/g in Madi samples to 58.17 ± 1.52 mg CE/g in Dharampur samples. For the fruiting stage, the content ranged from 17.54 ± 0.42 mg CE/g in Lad badhol samples to 37.34 ± 0.97 mg CE/g in Dharampur samples. (Sindhu and Arora, 2012) also indicated a similar flavonoid content of 43.58 ± 1.89 mg CE/g.

This increase in the phenolic and flavonoid content from pre-flowering to flowering stage and their subsequent decrease at fruiting stage was also observed by (Kasera et al., 2018) in medicinal plants namely Withania coagulans, Dipcadi erythraeum, Corbichonia Decumbens and Arisaema tortuosum. Vlaisavljević et al. (2017) stated that variations in phenolics and flavonoids during transitions from one growth phase to another is fundamentally related to the changes in the biosynthetic pathways at the molecular level. These changes alter the synthesis, allocation and disintegration of these metabolites during developmental stages (Pretti et al., 2018). Further, the phenolic content can decrease as a result of the breakdown of secondary metabolites into simple intermediate metabolites like lignin (Shao et al., 2014). These findings relate to an earlier study that indicated that the phenolic and flavonoid contents were highest in the flowering stage and substantially decreased to half at the fruiting stages (Pinhatti et al., 2010; Ben Farhat et al., 2015; Oszmiański et al., 2018; Farhadi et al., 2020). For the pre-flowering stage, the tannins in MK leaves ranged from 75.75 ± 1.69 mg TAE/g in Dhira samples to 143 ± 3.74 in Dharampur samples. For the flowering stage, the tannin content ranged from 116 ± 3.26 mg TAE/g in Sagned samples to 207 ± 5.42 mg TAE/g in Dharampur samples. For the fruiting stage, the tannin content ranged from 47 ± 1.18 mg TAE/g in Mlan samples to 156 ± 4.05 mg TAE/g in Nagri samples. A similar content of 206.05 ± 7.50 mg TAE/g was reported by (Uraku and Nwankwo, 2015) and 0.86 ± 0.02 g/100 g by (Igara et al., 2016).

High flavonoid and tannin content in flowering stages was also confirmed in Ziziphora clinopodioides by (Ding et al., 2014), Nigella sativa by (Zribi et al., 2014). An increase in flavonoid concentration at the flowering stage could result could be attributable to the fact that flavonoids determine flower and fruit coloration, flower aroma, pigmentation, ultra-violet protection (Nishihara and Nakatsuka, 2011). More flavonoids and tannins are likely to be synthesized by the plant with the onset of flowering. An increase in biosynthesis of flavonoids and tannins during flowering could be a defense mechanism against a wide variety of pests that could attack the plants and could serve as chemical signals for attracting potential pollinators, oviposition stimulants, phytoalexins, feeding attractants, deterrents (Iwashina, 2003).

Phenylpropanoids like phenols, flavonoids, tannins are synthesized from the shikimate pathway. The first committed step for protein and phenolic synthesis is catalyzed by phenylalanine ammonia-lyase, using phenylalanine (an aromatic amino acid) as a precursor (Yadav et al., 2020). The protein competition model states that the competition between proteins and phenolic synthesis for the common limiting phenylalanine, leads to a process-level tradeoff between the phenolic versus phenolic synthesis rates, establishing an inverse relation between phenolic and protein allocation (Jones and Hartley, 1999). Thus, when protein synthesis rates are high, the phenolic synthesis must be low and vice versa. Protein synthesis is increased during the pre-flowering and fruiting stage of plant development, while it is comparably less during the flowering stage (Frenkel et al., 1968). Thus a logical explanation could indicate that the high rates of protein synthesis during pre-flowering and fruiting stages could channelize the phenylalanine and carbon sources toward proteins synthesized, compromising the phenylpropanoid biosynthesis. Similarly lower protein synthesis during the flowering stages could enable the carbon flow toward the biosynthesis of phenols and flavonoids. Studies also indicate that these compounds have important roles in adaptation and providing defense against pathogens, herbivores and other environmental stresses during the critical flowering stages (Akula and Ravishankar, 2011).

For the pre-flowering stage, the essential oils in MK leaves ranged from 0.64 ± 0.01% in samples obtained from Mlan to 0.89% ± 0.02% in Dhramn samples. For the flowering stage, the content ranged from 0.85 ± 0.02% in Malghota samples to 1 ± 0.02% Dharampur samples. For the fruiting stage, the content ranged from 0.54 ± 0.01% in Lad badhol samples to 0.7% ± 0.01% in Dharampur samples. A similar essential oil yield of 0.24% was reported by (Rana et al., 2004) and 0.12%–0.18% by (Verma et al., 2012). Castelo et al. (2012) stated that the essential oil production was enhanced during the flowering period to attract pollinators such as bees and other insects.

Quinones are oxidized derivatives of aromatic compounds readily made from reactive aromatic compounds with electron-donating substituents such as phenols and catechols. Quinones are a group of compounds that are majorly found in the cell membranes of living organisms. They have a hydrophilic head and non-polar isoprenoid side chain, giving these molecules lipid-soluble properties. Quinones primarily function as proton and electron carriers in respiratory and photosynthetic electron transport chains, with additional antioxidant functions (Nowicka and Kruk, 2010). For the pre-flowering stage, the quinone in MK leaves ranged from 2.05 ± 0.05 mM/min/g tissue in Madi samples to 2.97 ± 0.07 mM/min/g tissue in Tang samples. For the flowering stage, the quinone content ranged from 3.07 ± 0.07 mM/min/g tissue in Kunnu samples to 4.95 ± 0.13 mM/min/g tissue in Dharampur samples. For the fruiting stage, the content ranged from 1.02 ± 0.02 mM/min/g tissue in Pudva samples to 1.96 ± 0.04 mM/min/g tissue in Neri samples.

For the pre-flowering stage, the antioxidant activity in MK leaves ranged from 4.01 ± 0.09 μg/ml in Kunnu samples to 7.42 ± 0.17 μg/ml in Malghota samples. For the flowering stage, the content ranged from 8.08 ± 0.19 μg/ml in Dhramn samples to 13.60 ± 0.35 μg/ml in Dharampur samples. For the fruiting stage, the content ranged from 3.11 ± 0.06 μg/ml in Dhira samples to 6.37 ± 0.15 μg/ml in Lad badhol. The high amount of antioxidant activities during flowering could be attributable to the higher content of phenols, flavonoids, quinones, tannins and essential oils during these growth stages. Studies indicate that phenols, flavonoids, tannins, and quinones have a distinct property to scavenge reactive oxygen species (ROS), which encompass nonradical oxygen species (HOCl, ^1^O_2_, H_2_O_2_, NO^•^, HO^•^, O_2_^–•^), radical oxygen species and oxidatively generated free radicals like ROO^•^ and RO^•^ like those created through oligonucleic acids (RNA and DNA), proteins, low-density lipoproteins (LDLs; Quideau et al., 2011). These compounds also display their antioxidant activities by chelating metal ions like iron(III)/copper(II) and iron(II)/copper(I) which mediate the conversion of O_2_^–•^ and H_2_O_2_ into highly reactive HO^•^ *via* Haber-Weiss/Fenton-type reactions (Andjelković et al., 2006). They also inhibit the activities of enzymes generating O_2_^–•^ like protein kinase C and xanthine oxidase (Pietta, 2000). These compounds exhibit their antioxidant activities by two main mechanisms. First is their ability to donate a hydrogen atom to free radicals, making the phenolic compounds as free radicals. The second mechanism is through single-electron transfer to a free radical forming a stable radical cation.

### Correlation analysis

The correlation coefficient was studied among phenols, flavonoids, antioxidant activity, essential oils, tannins, and quinones. Pearson’s correlation test was used for determining significant correlations among these parameters. Pearson’s r > 0 at *p* < 0.05 indicate significant positive correlation, while r < 0 at p < 0.05 indicate significant negative correlations. The values of Pearson’s r are indicated through a heat map in Figure 5, and their precise values are indicated in Supplementary Table S4. Antioxidant activity showed significant positive correlation with phenol content (r = 0.536; *p* < 0.001), essential oils (r = 0.707; *p* < 0.001), tannins (r = 0.564; *p* < 0.001), quinones (r = 0.820; *p* < 0.001), and flavonoids (r = 0.540; *p* < 0.001). As already mentioned, these highly significant positive correlations arise due to the ability of these compounds to reduce cellular oxygen concentration, intercept singlet oxygen (^1^O_2_) production, block the reaction chain initiation by scavenging hydroxyl radicals, chelate metal ions, terminate free radicals. These antioxidant activities rely on the unique structure and hydroxyl group arrangement in these compounds (Shahidi and Ambigaipalan, 2015). It is now clearly indicated by various studies that these phytochemicals have lower reduction potential than that of a free radical (oxidized species), thus they can instantaneously transfer their hydrogen atom to the free radical (Neudörffer et al., 2006; Quideau et al., 2011). Phenols showed significant positive correlations with flavonoids (r = 0.734; *p* < 0.001), tannins (r = 0.857; *p* < 0.001), quinones (r = 0.535; *p* < 0.001). This could be because all these compounds originate from the same polyketide and/or shikimate-derived phenylpropanoid pathway(s). Also, essential oils were positively correlated with phenols (r = 0.513; *p* < 0.001) and flavonoids (r = 0.470; *p* = 0.001) since the pathways of terpene (primary component of essential oils) biosynthesis (mevalonic acid and methylerythritol phosphate pathway) and phenol biosynthesis (malonic acid pathways and shikimic acid pathway) are interconnected through pyruvate and acetyl CoA (Mijts and Schmidt-Dannert, 2003; Mahajan et al., 2020). Thus, the increase in the synthesis of phenols, upregulates the terpene biosynthesis, subsequently increasing the essential oil content. Essential oils also indicated a significant positive correlation with antioxidant activity (r = 0.707; *p* < 0.001). This could be because of the strong peroxide decomposition and free radicals scavenging activities of terpenes present in the MK leaf essential oils (Bhavaniramya et al., 2019; Liang et al., 2021).

## Conclusion

The present study indicated the evidence that geographical locations and plant developmental stages primarily determine the total phenolics, flavonoids, essential oils, quinones, tannins and antioxidant activity in MK leaves. All evaluated phytochemical parameters significantly increased during the growth transition from pre-flowering to the flowering stage, followed by their gradual decrease during the fruiting stage. MVDA tools like PCA and HCA were used for visualizing the photochemical composition of MK leaves at different growth stages. HCA classified the samples into four different clusters based on their phytochemistry at three different developmental stages. PCA indicated that quinones, essential oils and antioxidant activity primarily determined the variability in the data. The study suggests that for utilizing the maximum phytochemical constituents, biological potency and industrial utility, MK leaves should be harvested at the flowering time. These phytochemicals can be used as raw materials in pharmaceuticals, cosmetics, insecticides, dyes, fragrances, nutraceuticals, etc. This study also establishes MVDA as an effective statistical tool to meaningfully decipher the phytochemical diversity in MK leaves. The information can significantly contribute toward the use of MK leaves aromatic and phytopharmaceutical industries.

## Data availability statement

The original contributions presented in the study are included in the article/Supplementary material, further inquiries can be directed to the corresponding author.

## Author contributions

RV and NS designed the work. RV collected and analyzed the samples. RV and AR executed parts of the experiments in the laboratory. MT, RB, and DD statistically analyzed the research data. RV and MT drafted and revised the manuscript. All authors contributed to the article and approved the submitted version.

## Funding

This work was supported by the Department of Chemistry and Biochemistry, Chaudhary Sarwan Kumar Himachal Pradesh Agriculture University, Palampur, HP (India).

## Conflict of interest

The authors declare that the research was conducted in the absence of any commercial or financial relationships that could be construed as a potential conflict of interest.

## Publisher’s note

All claims expressed in this article are solely those of the authors and do not necessarily represent those of their affiliated organizations, or those of the publisher, the editors and the reviewers. Any product that may be evaluated in this article, or claim that may be made by its manufacturer, is not guaranteed or endorsed by the publisher.

## Supplementary material

The Supplementary material for this article can be found online at: https://www.frontiersin.org/articles/10.3389/fpls.2022.963150/full#supplementary-material
