# Supplementary material for: Deciphering the growth stage specific bioactive diversity patterns in Murraya koenigii (L.) Spreng. using multivariate data analysis
Source: Front Plant Sci. 2022 Aug 25;13:963150. doi: 10.3389/fpls.2022.963150 (PMC9452700; doi:10.3389/fpls.2022.963150)
Supplement: Supplementary file 1 [file Data_Sheet_1.docx]

**Supplementary Table S1:** Bartlett's test for sphericity for phenols, flavonoids, antioxidants, essential oils, tannins, and quinones

| **χ²** | **df** | **p** |
| --- | --- | --- |
| 221 | 15 | < 0.001 |

**Supplementary Table S2:** KMO Measure of Sampling Adequacy for phenols, flavonoids, antioxidants, essential oils, tannins, and quinones

|  | **MSA** |
| --- | --- |
| **Overall** | 0.744 |
| Phenols | 0.665 |
| Flavonoids | 0.727 |
| Antioxidant activity | 0.850 |
| Essential oils | 0.799 |
| Tannins | 0.702 |
| Quinones | 0.743 |

**Note:** MSA (Measure of Sampling Adequacy)

**Supplementary Table S3:** Factor loadings for phenols, flavonoids, antioxidants, essential oils, tannins, and quinones

| **Component** | | | | |
| --- | --- | --- | --- | --- |
|  | **1** | **2** | **3** | **Uniqueness** |
| **Quinones** | 0.911 | 0.242 | 0.215 | 0.0649 |
| **Essential oil** | 0.882 | 0.274 | 0.126 | 0.1307 |
| **Antioxidant activity** | 0.816 | 0.247 | 0.294 | 0.1863 |
| **Tannins** | 0.340 | 0.910 | 0.156 | 0.0320 |
| **Phenols** | 0.251 | 0.809 | 0.484 | 0.0492 |
| **Flavonoids** | 0.282 | 0.304 | 0.902 | 0.0147 |

**Supplementary Table S4:** Pearson's correlation values between phenols, flavonoids, antioxidants, essential oils, tannins and quinones

|  |  | **Phenols** | **Flavonoids** | **Antioxidant activity** | **Essential oils** | **Tannins** | **Quinones** |
| --- | --- | --- | --- | --- | --- | --- | --- |
| **Phenols** | Pearson's r | — |  |  |  |  |  |
|  | p-value | — |  |  |  |  |  |
| **Flavonoids** | Pearson's r | 0.734^***^ | — |  |  |  |  |
|  | p-value | < 0.001 | — |  |  |  |  |
| **Antioxidant activity** | Pearson's r | 0.536^***^ | 0.540^***^ | — |  |  |  |
|  | p-value | < 0.001 | <0.001 | — |  |  |  |
| **Essential oils** | Pearson's r | 0.513^***^ | 0.470^**^ | 0.707^***^ | — |  |  |
|  | p-value | < 0.001 | 0.001 | < 0.001 | — |  |  |
| **Tannins** | Pearson's r | 0.857^***^ | 0.527^***^ | 0.564^***^ | 0.552^***^ | — |  |
|  | p-value | < 0.001 | < 0.001 | < 0.001 | < 0.001 | — |  |
| **Quinones** | Pearson's r | 0.535^***^ | 0.524^***^ | 0.820^***^ | 0.873^***^ | 0.560^***^ | — |
|  | p-value | < 0.001 | < 0.001 | < 0.001 | < 0.001 | < 0.001 | — |

**Note:** * p < 0.05, ** p < 0.01, *** p < 0.001
