# Supplementary material for: Deciphering the growth stage specific bioactive diversity patterns in Murraya koenigii (L.) Spreng. using multivariate data analysis
Source: Front Plant Sci. 2022 Aug 25;13:963150. doi: 10.3389/fpls.2022.963150 (PMC9452700; doi:10.3389/fpls.2022.963150)

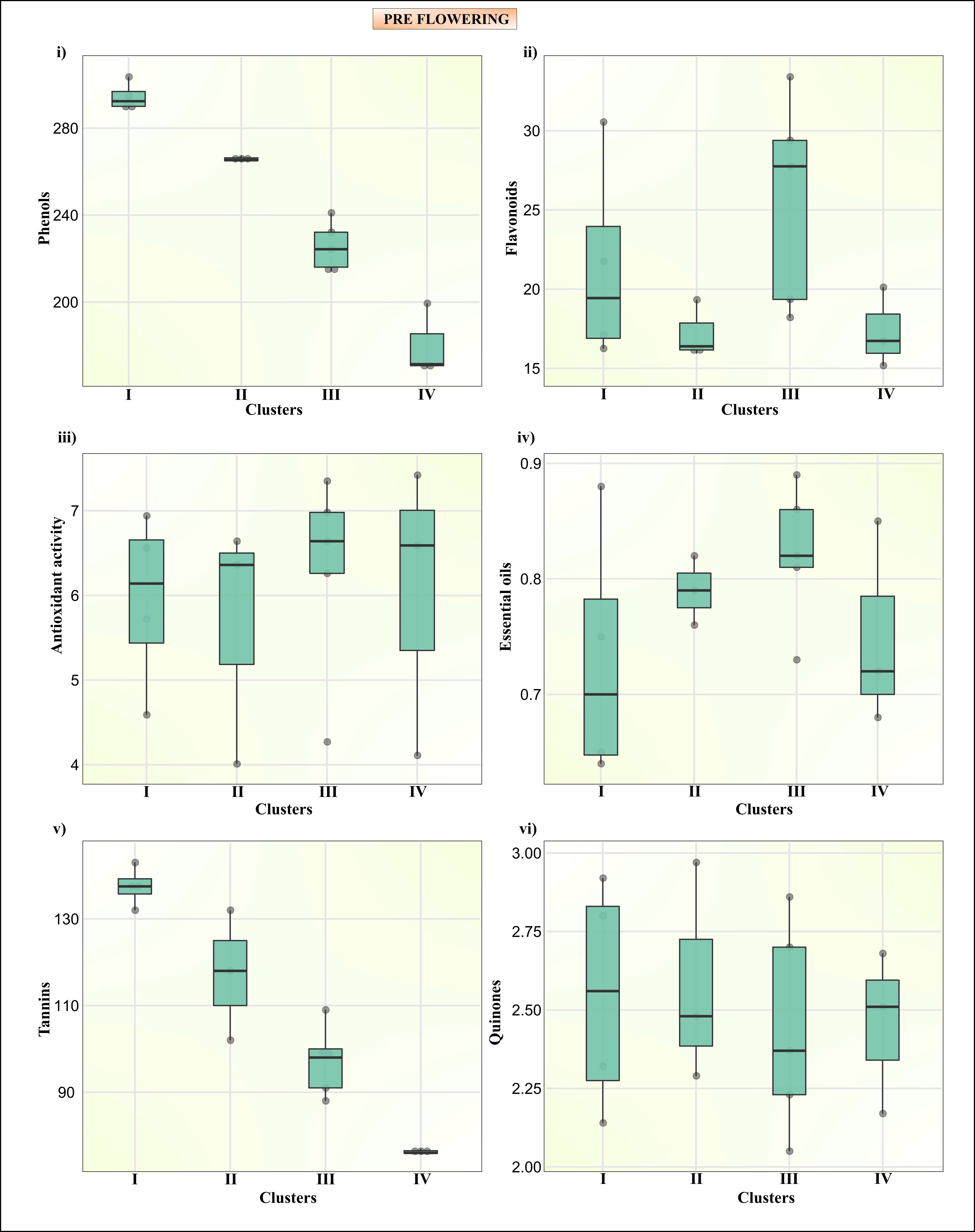
**Fig. S1:** Mean values of phenols, flavonoids, antioxidants, essential oils, tannins and quinones for each cluster at pre-flowering stage indicated through by Box-and-whisker plots.

**Fig. S2:** Mean values of phenols, flavonoids, antioxidants, essential oils, tannins and quinones for each cluster at flowering stage indicated through by Box-and-whisker plots.


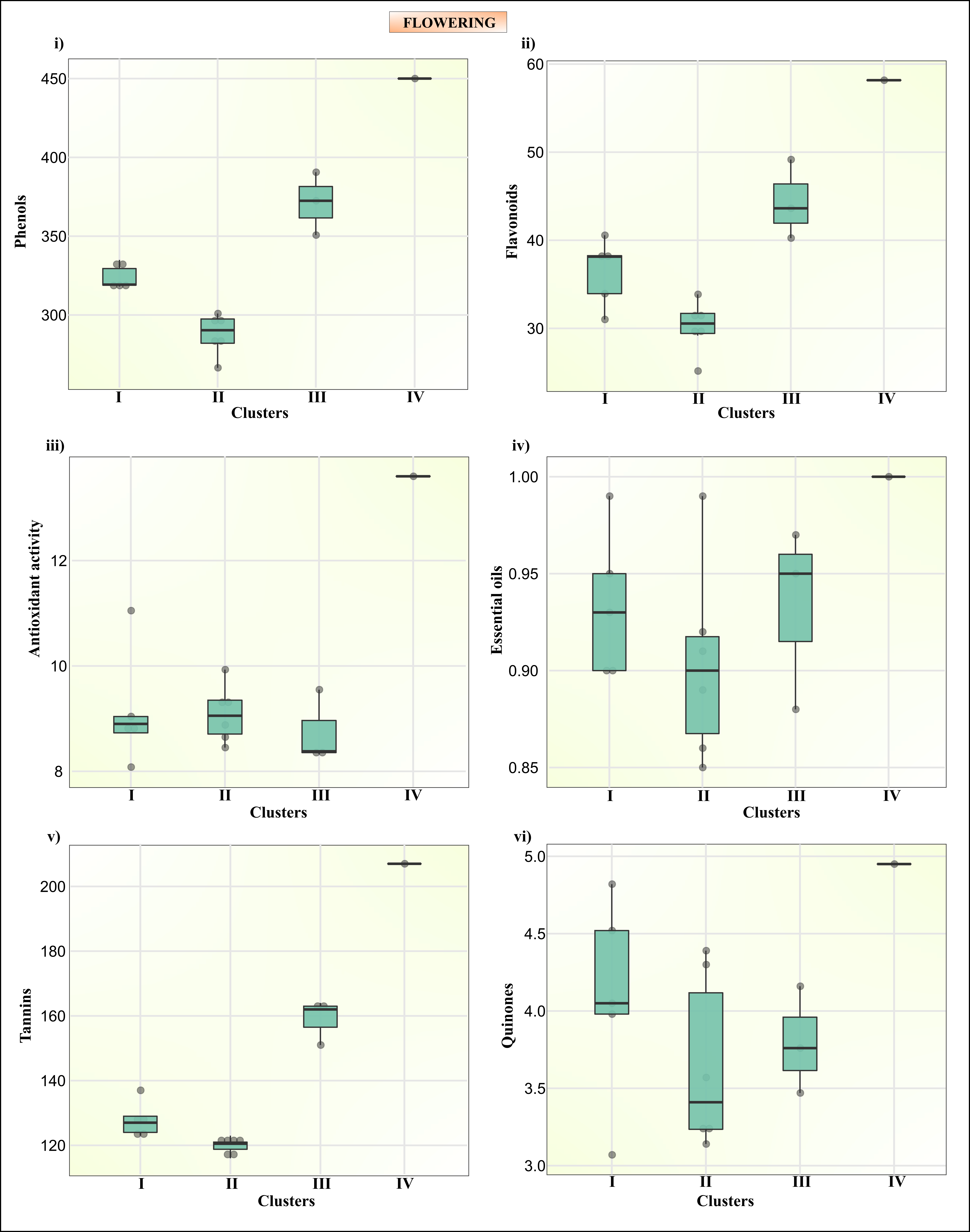


**Fig. S3:** Mean values of phenols, flavonoids, antioxidants, essential oils, tannins and quinones for each cluster at fruiting stage indicated through by Box-and-whisker plots.


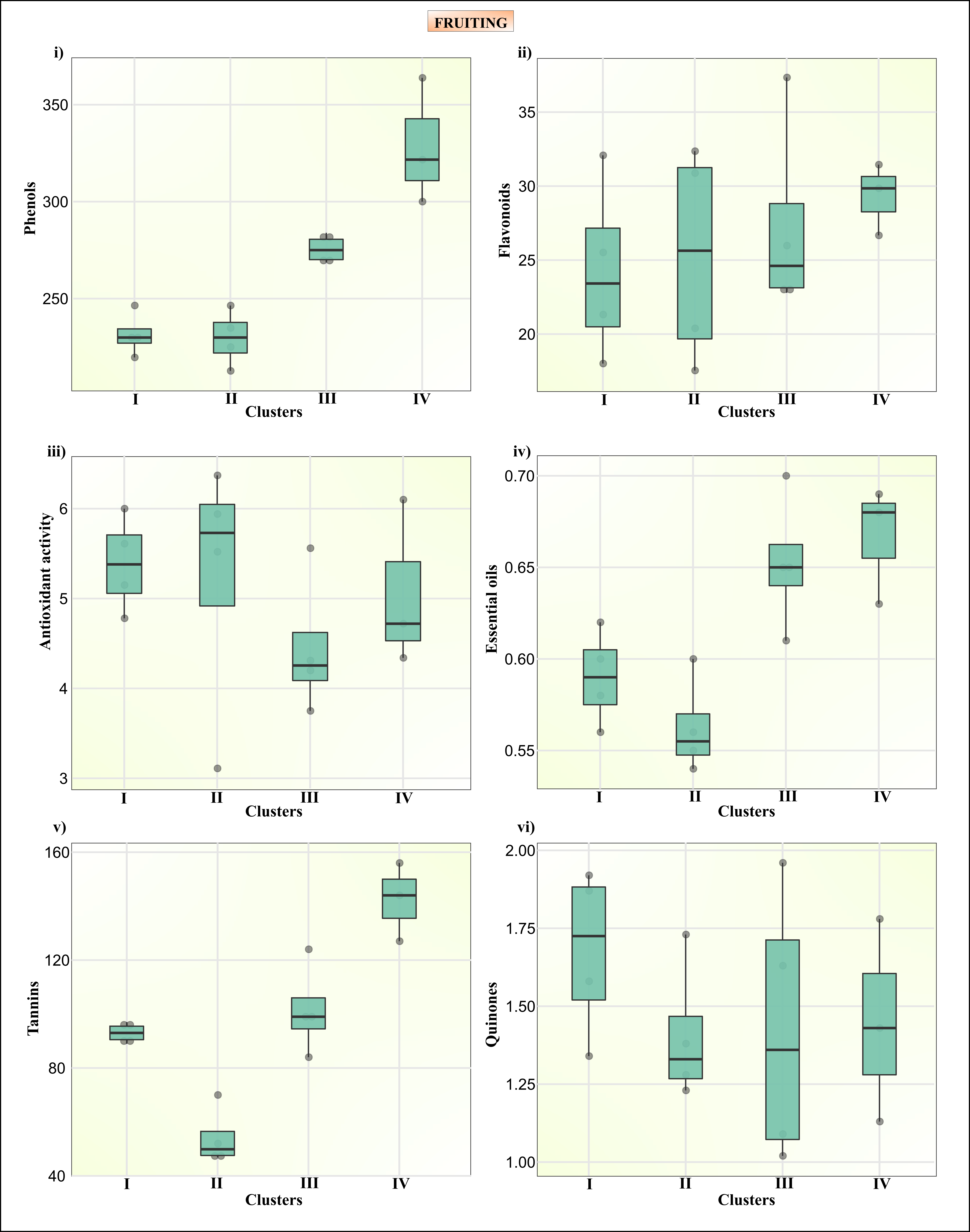


**Fig. S4:** Mean values of phenols, flavonoids, antioxidants, essential oils, tannins and quinones at each developmental stage indicated through bar graphs.


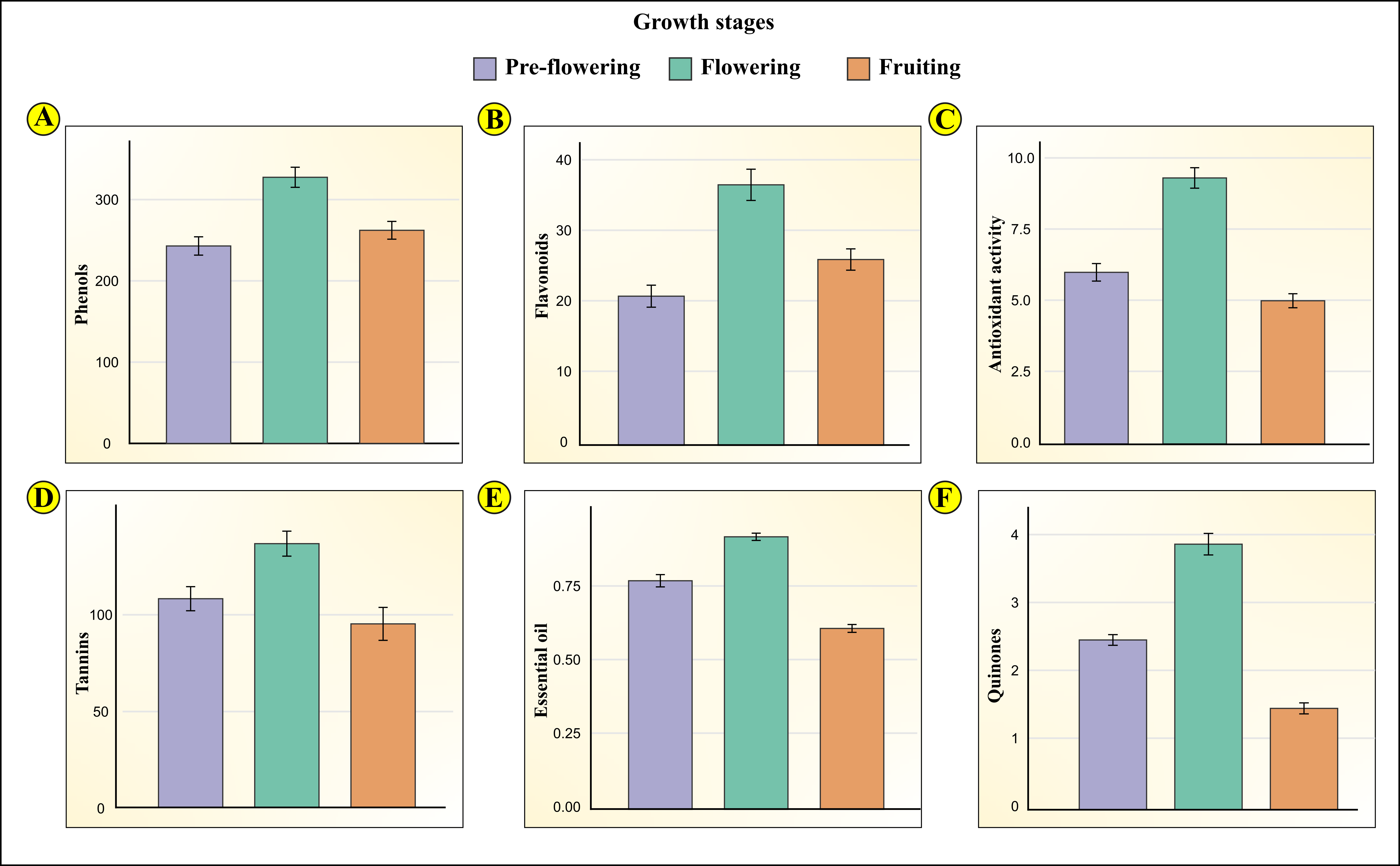

Supplement: Supplementary file 2 [file Data_Sheet_2.docx]
